# The Cutaneous Manifestations of Drug Reactions Can Mimic Traumatic Injuries: Case Reports and the Potential Role of Forensic Dermatology

**DOI:** 10.7759/cureus.47734

**Published:** 2023-10-26

**Authors:** Philip R Cohen

**Affiliations:** 1 Dermatology, University of California Davis Medical Center, Sacramento, USA; 2 Dermatology, Touro University California College of Osteopathic Medicine, Vallejo, USA

**Keywords:** trauma, reaction, pathology, mimic, injuries, forensic, drug, dermatology, cutaneous, abuse

## Abstract

The evaluation of the skin of the decedent is an essential component of the assessment by the forensic pathologist or the medical examiner. Age-associated cutaneous changes, primary diseases of the skin, and systemic conditions with mucocutaneous manifestations can be present. Importantly, several dermatoses can be misinterpreted for traumatic injuries; specifically, adverse reactions to medications can mimic assault, burns, elder abuse, and mutilation or torture. A male with corticosteroid-induced dermatitis mimicking an acute burn is described. A female with thalidomide embryopathy is reported with extensive deformities of her hands and feet with multiple absent digits mimicking a severe injury resulting from mutilation or torture. Another female is described who had hydroxychloroquine-associated hyperpigmentation; her physician misinterpreted the cutaneous hyperpigmentation as bruises and notified Adult Protective Services. Reactions to medications can also mimic assault, burns, and elder abuse. Drug reaction with eosinophilia and systemic symptoms (particularly when associated with phenytoin) can mimic assault. Albeit rarely, the antihypertensive irbesartan can result in dramatic edema of the face and eyelids similar to that observed following an assault. Drug-induced erythema multiforme can mimic a localized burn, and Stevens-Johnson syndrome or vancomycin infusion reaction can mimic an extensive burn. Several medications can mimic bruising observed in victims of elder abuse; they include amiodarone, arsenic, and tetracyclines (such as minocycline and doxycycline). In summary, an important aspect of the forensic evaluation during an autopsy includes a complete cutaneous examination; to aid in differentiating medication-associated dermatoses that can mimic traumatic injury, the evaluation of the decedent by a forensic dermatologist may be helpful to establish the etiology of observed skin changes.

## Introduction

Forensic pathologists, medical examiners, and physicians in general need to be able to differentiate abuse from other etiologies. Several dermatologic conditions can mimic abuse to a child or an adult [1,2]. Drug reactions can have similar manifestations to the features observed in victims who have been abused [3].

Drug reactions can result from topical medications. They can also occur from systemic medications received by the patient. In addition, albeit less common, they can occur in patients whose mother was exposed to the drug during gestation [4-6].

A male with dermatitis induced by exposure from the application of a potent corticosteroid cream that mimicked a burn to the forehead is presented. A female, whose mother had received thalidomide during pregnancy, with distal limb defects that mimicked severe trauma or a mutilating injury is reported. In addition, a female with hydroxychloroquine-associated blue discoloration of her skin that mimicked elder abuse is described. A complete cutaneous examination is an essential component of the forensic evaluation at an autopsy; indeed, a forensic dermatologist may be able to assist in the differentiation of cutaneous manifestations caused by medication that can mimic abuse [7].

## Case presentation

Case 1

A male in his late 50s presented with a bright red confluent area on his forehead and red cheeks with occasional pimples. He had no prior history of sun sensitivity or excessive sun exposure. After an initial inspection, prior to obtaining a medical history, the clinical differential diagnosis of his forehead lesion included a localized superficial burn. He had a tube of Gelmicin cream that he had been applying two to three times daily to his face for at least six months. The medication consisted of a high-potency corticosteroid (betamethasone 0.05%), an antibiotic (gentamycin 0.1%), and an antifungal agent (clotrimazole 1.0%).

**Figure 1:**
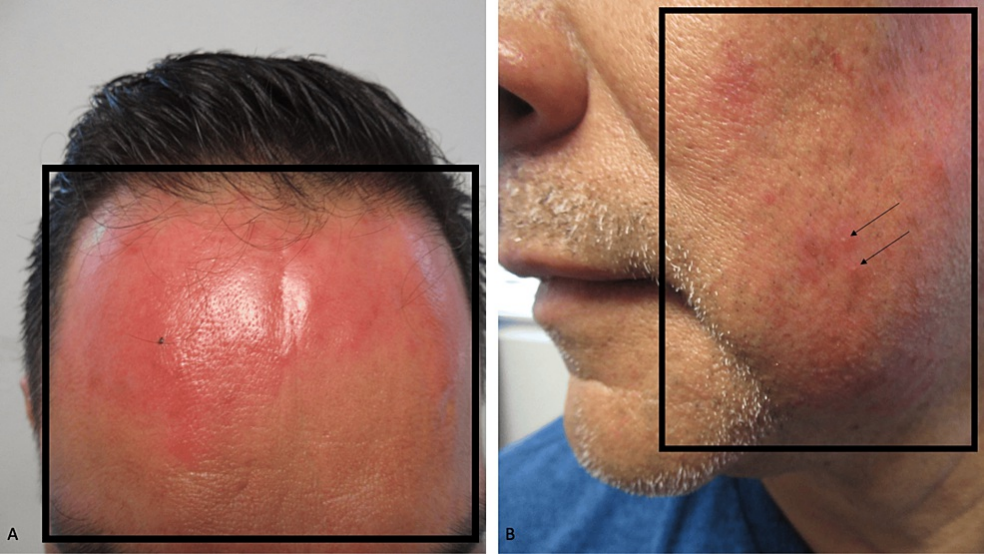
Topical corticosteroid-induced dermatitis mimicking an acute burn. Frontal view of the forehead (A) and lateral view of the left cheek (B) of a male in his late 50s who applied the high-potency betamethasone 0.05% cream several times a day to his forehead and cheeks. A patch of confluent erythema on the forehead, which mimics a superficial or first-degree burn, is outlined by the black rectangle (A). Macular erythema, outlined by the black rectangle, is shown on the left cheek (B); occasional pustules, a characteristic of dermatitis caused by topical high-potency corticosteroid applied to the face, are also present (black arrows).

The cutaneous examination of his face showed an erythematous macular patch that occupied nearly his entire forehead. There was erythema on his cheeks. Occasional randomly distributed pustules were present on the cheeks (Figure 1).

The correlation of the clinical history and morphologic appearance of the lesions established a diagnosis of corticosteroid-induced dermatitis. This patient had more pronounced clinical involvement that included the areas not only around his mouth but also his cheeks and forehead. The possibility of a localized burn to his forehead was excluded.

Treatment included oral doxycycline 100 mg twice daily and progressive tapering of the potency and frequency of the topical corticosteroid. He progressively improved, and both the oral antibiotic and low-potency corticosteroid being applied were decreased to once daily. After three months, he was no longer applying any corticosteroid cream to his face, and the doxycycline was stopped. After three additional months of follow-up, there was no recurrence.

Case 2

**Figure 2:**
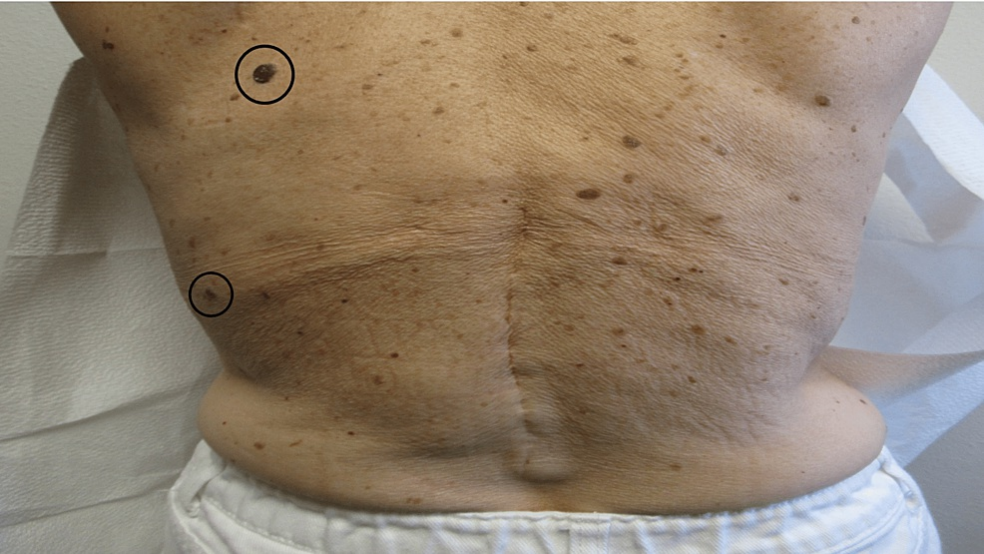
Seborrheic keratoses on the back presenting as dark plaques. The back of a female in her 60s has numerous dark plaques. The size and color of two of the lesions (within black ovals) had changed. The microscopic examination of the biopsy specimens from both plaques each showed a benign irritated seborrheic keratosis. The image has not been previously published.

A female in her 60s presented for the evaluation of two dark plaques on her back that had changed in size and color (Figure 2). Both lesions were conservatively removed using the shave biopsy technique. The microscopic examination of the specimens demonstrated completely removed irritated seborrheic keratoses.

**Figure 3:**
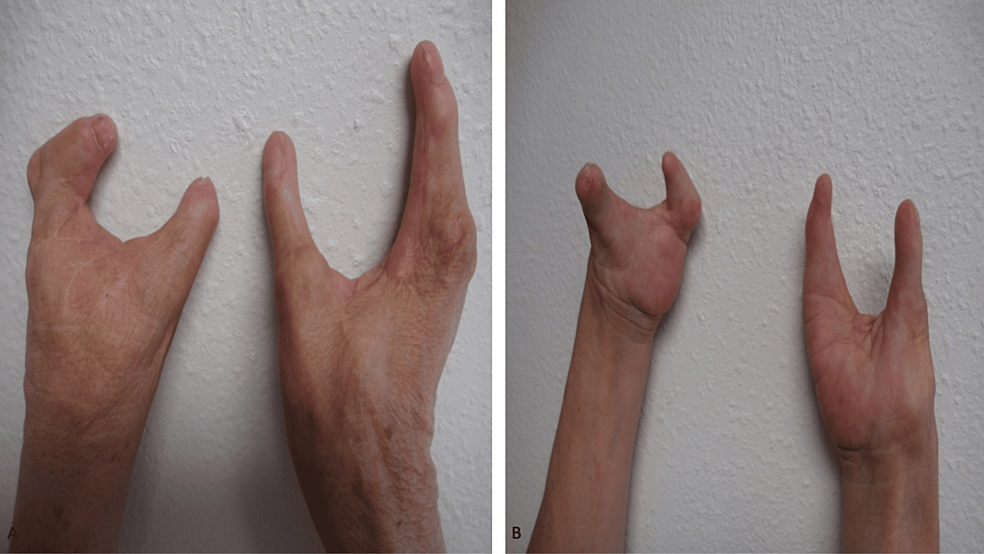
Thalidomide embryopathy affecting the upper extremities mimics a mutilation injury. The dorsal view (A) and the palmar view (B) of the right and left hand of a female whose mother had been exposed to thalidomide during gestation. The images have not been previously published.

**Figure 4:**
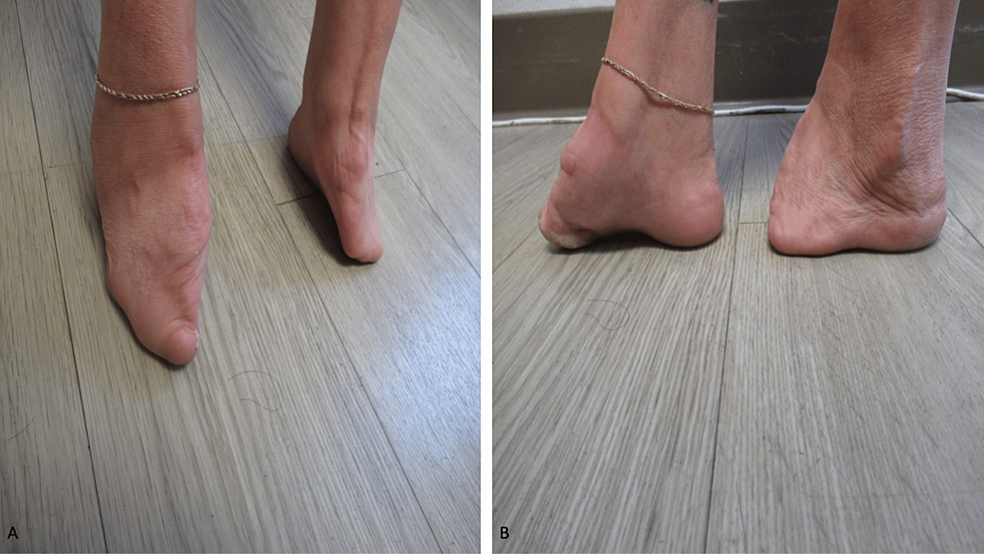
Thalidomide embryopathy involving the feet mimics injury secondary to severe torture. The frontal (A) and lateral (B) views of the right and left foot show an absence of multiple digits; the female’s mother had been treated with thalidomide during pregnancy. The images have not been previously published.

A complete skin examination was performed. Inspection revealed severe abnormalities of both the distal upper extremities and lower extremities (Figures 3, 4). This was her first visit to see me, and my initial impression was that the defects were compatible with either a severe injury or mutilating torture. Additional history was obtained; her mother had been exposed to thalidomide during pregnancy.

Only two digits were present on each hand. Most of the toes were absent on both feet. She also had strabismus, a high-arched palate, a deformity of both external ears, and a hypermobility of her hips. The correlation of her medical history and clinical examination demonstrated several features of thalidomide embryopathy. Some of the details of this patient have been previously reported [5].

Case 3

A female in her 60s presented for an evaluation of her skin. Her past medical history was significant for myasthenia gravis, which was currently being managed with plasmapheresis. She also had systemic lupus erythematosus; she has been treated with 400 mg daily of hydroxychloroquine for more than 30 years.

**Figure 5:**
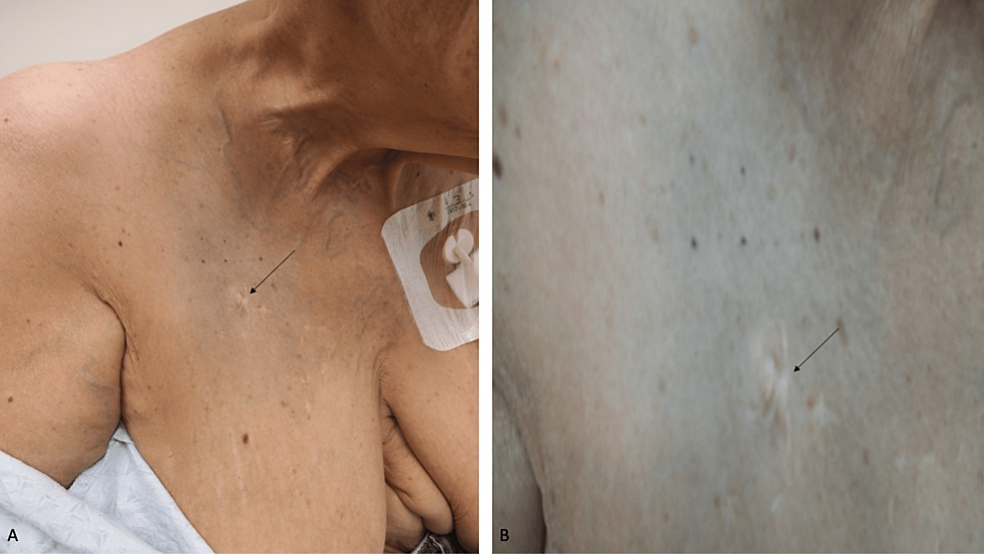
Hydroxychloroquine-related cutaneous hyperpigmentation mimicking elder abuse-associated bruising. The distant (A) and closer (B) view of the chest of a 66-year-old female who had been taking hydroxychloroquine for more than 30 years to treat systemic lupus erythematosus. The access line for her plasmapheresis treatment of myasthenia gravis is inserted on the left side of her chest (A). The right side of her chest has blue dyschromia mimicking bruising (A and B); the healed scar from a prior access line is demonstrated by the black arrows. The patient’s family was investigated by Adult Protective Services and eventually cleared of the elder abuse allegations that were made by a physician who misinterpreted her skin color changes to be bruising. The images have not been previously published.

**Figure 6:**
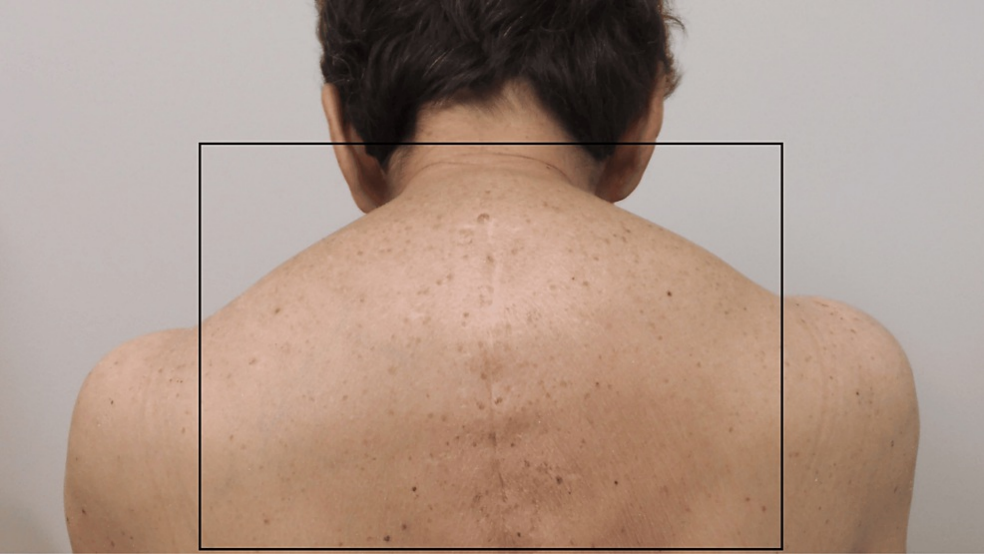
Diffuse cutaneous hyperpigmentation mimicking elder abuse-associated bruising by hydroxychloroquine. The entire upper back shows diffuse black hyperpigmentation (outlined by the black rectangle) associated with chronic hydroxychloroquine therapy that mimicked bruising, which was misinterpreted as elder abuse. The image has not been previously published.

Cutaneous examination showed diffuse hyperpigmentation of her face, neck, chest, and back. The right upper chest was blue (Figure 5). The upper back was black (Figure 6).

Additional history was shared by the patient and her family. Five years earlier, her hydroxychloroquine-related cutaneous hyperpigmentation was misinterpreted after she was seen by a new clinician. The physician contacted Adult Protective Services to report that the patient was a victim of elder abuse and had suffered extensive bruising. A few days after the appointment, a worker from the agency arrived at the patient’s home to investigate the family. Eventually, the family was cleared of the elder abuse allegations. Some of the details of this patient have been previously reported [6].

## Discussion

A thorough cutaneous examination performed by a forensic specialist is recommended in highly suspicious cases [8]. The awareness of age-related skin alterations and dermatology-associated diseases by the investigator can prevent unnecessary forensic investigation and stress to the family of the dead person. In addition to postmortem artifacts, dermatoses can be mistaken for traumatic injuries [3,9].

Adverse effects from medications can result in dermatologic manifestations that affect not only the skin and mucosal membranes but also the hair and nails. Mucocutaneous drug reactions can result from both topical and systemic administrations of the agents. These drug reactions can mimic traumatic injuries (Table 1) [3-6,10-19]. The three patients described in this article developed cutaneous drug reactions that mimicked traumatic injuries; the assessment of their clinical features, without obtaining additional medication history, might have resulted in the misinterpretation of their etiology.

**Table 1:** Drug associated with potentially being mistaken for traumatic injuries CR, current report; DRESS, drug reaction with eosinophilia and systemic symptoms; EM, erythema multiforme; SJS, Stevens-Johnson syndrome; TEN, toxic epidermal necrolysis; TMP-SMX, trimethoprim-sulfamethoxazole

Misdiagnosed traumatic injury	Drug	Comment	References
Assault	DRESS-associated phenytoin	Severe edema and swelling of the entire face can mimic postassault swelling. This has previously been referred to as drug-induced hypersensitivity syndrome; when associated with phenytoin, it had been known as anticonvulsant hypersensitivity syndrome. It can also be caused by other anticonvulsants and drugs. The onset is within three weeks to three months after starting the drug; there is fever, lymphadenopathy, and at least one internal organ system involved, and blood abnormalities may include lymphocytosis, eosinophilia, and thrombocytopenia.	[10,11]
Assault	Irbesartan	A 52-year-old female developed progressive and sustained puffiness of her eyelids, face, and neck within two months after starting the drug; the presentation of her face mimicked the appearance of having been assaulted. The severe edema did not improve after stopping the drug. She elected to again take the drug to control her blood pressure; the edema remained unchanged two years later.	[12]
Burn, localized	Corticosteroids, topical	The 59-year-old male’s forehead was bright red, and the erythema mimicked an acute localized burn. His skin changes resulted from the repeated application of an ultrapotent corticosteroid (betamethasone).	[4] and CR
Burn, localized	EM-associated ampicillin	EM can result from not only exposure to several different drugs (such as antibiotics including penicillins and sulfonamides, antiepileptics, and nonsteroidal anti-inflammatory drugs) but also infections such as Mycoplasma pneumoniae and herpes simplex virus (types 1 and 2). The skin lesions can mimic a localized burn; in addition, the lesions may be targetoid and affect the mucous membranes of the anus, eyes, mouth, and/or genitalia.	[3,13]
Burn, extensive	SJS-TEN-associated TMP-SMX	SJS-TEN are severe cutaneous adverse reactions; the body surface area affected by SJS is less than 10% and more than 30% by TEN. They can mimic an extensive scalded burn; there can be peeling of the skin. In contrast to a burn, the hair is not singed in SJS-TEN. In addition to TMP-SMX and other antibiotics, other drug triggers of SJD-TEN are allopurinol, antiepileptics, immune checkpoint inhibitors (such as nivolumab and pembrolizumab), nevirapine, and nonsteroidal anti-inflammatory drugs.	[3,11,14]
Burn, extensive	Vancomycin	Vancomycin infusion reaction was previously referred to as vancomycin flushing syndrome and originally as red man syndrome; the latter term was considered to have potential gender bias and race-related bias. The infusion reaction can occur following intravenous administration of the drug; in addition, receiving the drug orally or intraperitoneally during dialysis can also result in the hypersensitivity reaction. The patient develops a pruritic, confluent erythema predominantly affecting the upper body, which can be accompanied by wheezing and hypotension. The large red rash can mimic an extensive burn.	[15]
Elder abuse, bruise	Amiodarone	Amiodarone can cause blue-gray hyperpigmentation in sun-exposed sites; this appears after 40 g of therapy and usually after at least four months of treatment. The skin discoloration can mimic a bruise.	[16]
Elder abuse, bruise	Arsenic	Chronic arsenic toxicity can be associated with various presentations of pigmentary changes. In addition to leukomelanosis (depigmented macules), hyperpigmentation can appear as spotted melanosis or raindrop pigmentation, dyschromia (with hyperpigmented and hypopigmented macules), and diffuse melanosis that mimics bruising. Chronic arsenic toxicity many include mucosal pigmentation (of the tongue, gums, or buccal mucosa) and transverse white bands (Mees lines) on the fingernails.	[17]
Elder abuse, bruise	Plaquenil	The 66-year-old female in this report had been taking hydroxychloroquine for systemic lupus erythematosus for more than 30 years; she developed blue dyschromia on her chest, which mimicked bruising, and her new physician contacted Adult Protective Services concerned that she was a victim of elder abuse.	[6] and CR
Elder abuse, bruise	Tetracyclines	Not only minocycline but also doxycycline can cause blue-black hyperpigmentation that mimics bruising. The medications can be used for the long-term treatment of acne, rosacea, and infections. Three types of hyperpigmentation associated with minocycline use include blue-black in areas of prior inflammation or scarring (type I); blue-gray in areas of previously healthy skin, such as the lower legs (type II); and diffuse muddy brown discoloration in sun-exposed areas (type III). Doxycycline pigmentation usually occurs on the face and at the site of a previous scar. In contrast to bruising, minocycline hyperpigmentation can occur on mucosal membranes such as the tongue and the subungual nail bed.	[18,19]
Mutilation, torture	Thalidomide	The 63-year-old female had absent fingers on both hands and absent toes on both feet. Her missing digits mimicked injury (by torture) or mutilation (by amputation); her history reveals that her mother had received thalidomide during her pregnancy. Other features of thalidomide embryopathy including strabismus, hypermobility of her hips, a high-arched palate, and external ear abnormalities were also present.	[5] and CR

Burns can result from various etiologies. The appearance of a sunburn on the skin can be similar to that of either a first-degree burn or a second-degree burn. In addition, not only chemical corrosives but also hot objects or scalding liquids contacting the skin can result in localized injury at the site of impact. Radiation and flames can cause more extensive burns [3,20].

Drug hypersensitivity reactions such as erythema multiforme and Stevens-Johnson syndrome-toxic epidermal necrolysis can mimic localized or extensive burns. In contrast to burns, new skin lesions associated with these adverse side effects from medications may continue to appear, and the mucosal involvement of the eyes, mouth, and genitalia may develop. Also, in burn injuries, the hair in the affected areas is often singed; yet, in drug reactions, the hair follicles may remain unchanged [3,11,13,14,20].

Generalized macular erythroderma simulating an extensive burn can be mimicked by the acute presentation of vancomycin infusion reaction. The reaction to vancomycin frequently resolves after the discontinuation of the causative drug and treatment with systemic corticosteroids and antihistamines; albeit less common, the skin changes can persist before eventually resolving [15].

Topical corticosteroid-associated perioral dermatitis typically appears on the skin around the mouth. It is caused by the repetitive topical application of a high-potency corticosteroid, often for a prolonged duration of application, at least once daily, to the affected areas on the face [4]. Similar to the male described in this report, his confluent forehead erythema mimicked a localized burn injury.

The clinical presentation of drug reactions of the skin can mimic the cutaneous stigmata observed in survivors of torture, physical abuse, or assault. Torture is usually prompted by the instigation of the assailant by a public official or a person acting as an authority figure; it involves severe pain and suffering and includes intentional physical or mental injury that is inflicted on an individual. Physical abuse and assault occur more commonly in elderly persons and children; indeed, the victims often know and trust their perpetrator [20].

Physical abuse also includes elder abuse. Dyschromia can be misinterpreted as elder abuse-associated bruising [3,6,20]. Pigmentary alteration can also result from toxic metal exposure, such as arsenic [17].

Drug-related hyperpigmentation can mimic a bruise. The clinical presentation of bruises created traumatically may reflect the shape of the instrument that was applied to the skin. In contrast, medication-associated hyperpigmentation often has indistinct borders; ecchymoses and erythema are typically absent [20].

Mucocutaneous hyperpigmentation can be observed in patients receiving tetracyclines. Minocycline-related hyperpigmentation may occur on healthy skin that has not been exposed to the sun. Doxycycline-induced skin darkening usually occurs on the face. Both drugs can result in the dyschromia of scars.

Skin darkening from minocycline can also occur in sun-exposed sites; amiodarone-associated hyperpigmentation only occurs in sun-exposed locations. The skin darkening resulting from these drugs can mimic a bruise; however, in some of the patients who develop drug-induced dyschromia from the tetracyclines, there is a concurrent darkening of either the subungual nail bed or the mucosa of the sclera, gums, or tongue [16,18,19].

Chronic exposure to arsenic can result in various presentations of skin darkening. The hyperpigmentation can not only be localized but also diffuse and mimic a bruise; in addition, the pigmentation of mucosal membranes can be observed. When present, horizontal white bands on the nail plates (Mees lines) may be helpful to differentiate arsenic-related hyperpigmentation of the skin from bruising; the detection of arsenic after the analysis of the hair and fingernail plates can confirm the diagnosis of arsenic exposure [17].

Hydroxychloroquine can result in the blue-black discoloration of the skin. In the female described in this report, who had been taking the medication for more than 30 years as a part of her treatment for systemic lupus erythematosus, the drug-induced dyschromia was misinterpreted by a clinician who had not previously treated her as a manifestation of elder abuse. The concerned physician suspected that her darkened skin demonstrated bruises and was unaware that the patient’s cutaneous hyperpigmentation had occurred as a side effect of her medication. The doctor contacted Adult Protective Services; thereafter, an unnecessary, extensive investigation of the patient’s family occurred. Eventually, the issue was resolved, and the family was cleared of all suspicion of elder abuse.

Assault to the face may present with dramatic swelling. Acute swelling of the face can occur after the initiation of antiepileptic drugs. Dramatic facial swelling, with marked edema of the eyelids, resulting from phenytoin-associated drug reaction with eosinophilia and systemic symptoms can mimic the morphologic appearance of a severe assault to the face [10,11]. Albeit less common, marked facial swelling has developed and persisted after the initiation of the antihypertensive agent irbesartan [12].

Mutilation can result from prior torture or a severe injury [8]. Missing digits from either the hands or feet or both are usually caused by amputation. The exposure of the patient’s mother to a teratogen during gestation, such as thalidomide, is another cause of limb abnormalities [5].

Drug-associated extensive limb defects, presenting with absent digits, are rarely encountered especially since the use of thalidomide by pregnant females was discontinued. The woman described in this report who had in utero exposure to thalidomide presented with devastating sequalae to her hands and feet, which mimicked the clinical presentation that might be observed in a survivor of mutilating torture or a severely traumatic injury. She also had other stigmata of thalidomide embryopathy, such as strabismus, the hypermobility of her hips, a high-arched palate, and deformities of her external ears.

## Conclusions

The forensic pathologist or the medical examiner evaluates the skin of the decedent during an autopsy. Cutaneous changes relating to aging, skin conditions, and mucosal and cutaneous findings of systemic disorders may be present. The presentation of not only several dermatoses but also adverse reactions to medications can be misinterpreted for traumatic injuries. Including the patients in this report, adverse reactions to medications can mimic assault (facial swelling from either phenytoin-related drug reaction with eosinophilia and systemic symptoms or irbesartan), burns (from erythema multiforme, Stevens-Johnson syndrome, or vancomycin infusion reaction), and elder abuse (from cutaneous hyperpigmentation caused by either amiodarone, arsenic, minocycline, or doxycycline). In conclusion, evaluation by a forensic dermatologist may be helpful when performing the complete cutaneous examination of a decedent during an autopsy to assist in identifying medication-associated dermatoses that can mimic traumatic injury.
